# Predictive ability of an expert-defined population segmentation framework for healthcare utilization and mortality - a retrospective cohort study

**DOI:** 10.1186/s12913-019-4251-6

**Published:** 2019-06-20

**Authors:** Lian Leng Low, Yu Heng Kwan, Cheryl Ann Ma, Shi Yan, Elian Hui San Chia, Julian Thumboo

**Affiliations:** 1 Department of Family Medicine & Continuing Care, Singapore General Hospital, 20 College Road, Singapore, 169856 Singapore; 2 Family Medicine, Duke-NUS Medical School, Singapore, Singapore; 3 Singapore Heart Foundation, Singapore, Singapore; 4 Duke–NUS Medical School, Singapore, Singapore; 5 Office of Insights and Analytics, SingHealth, Singapore, Singapore; 6 Health Services Research Centre, Singapore Health Services, Singapore, Singapore; 7 SingHealth Regional Health System, Singapore Health Services, Singapore, Singapore; 8 Department of Rheumatology and Immunology, Singapore General Hospital, Singapore, Singapore

**Keywords:** Population segmentation, Healthcare utilization, Patient segmentation

## Abstract

**Background:**

Population segmentation of patients into parsimonious and relatively homogenous subgroups or segments based on healthcare requirements can aid healthcare resource planning and the development of targeted intervention programs. In this study, we evaluated the predictive ability of a previously described expert-defined segmentation approach on 3-year hospital utilization and mortality.

**Methods:**

We segmented all adult patients who had a healthcare encounter with Singapore Health Services (SingHealth) in 2012 using the SingHealth Electronic Health Records (SingHealth EHRs). Patients were divided into non-overlapping segments defined as Mostly Healthy, Stable Chronic, Serious Acute, Complex Chronic without Frequent Hospital Admissions, Complex Chronic with Frequent Hospital Admissions, and End of Life, using a previously described expert-defined segmentation approach. Hospital admissions, emergency department attendances (ED), specialist outpatient clinic attendances (SOC) and mortality in different patient subgroups were analyzed from 2013 to 2015.

**Results:**

819,993 patients were included in this study. Patients in Complex Chronic with Frequent Hospital Admissions segment were most likely to have a hospital admission (IRR 22.7; *p* < 0.001) and ED visit (IRR 14.5; *p* < 0.001) in the follow-on 3 years compared to other segments. Patients in the End of Life and Complex Chronic with Frequent Hospital Admissions segments had the lowest three-year survival rates of 58.2 and 62.6% respectively whereas other segments had survival rates of above 90% after 3 years.

**Conclusion:**

In this study, we demonstrated the predictive ability of an expert-driven segmentation framework on longitudinal healthcare utilization and mortality.

**Electronic supplementary material:**

The online version of this article (10.1186/s12913-019-4251-6) contains supplementary material, which is available to authorized users.

## Background

Population segmentation of patients into parsimonious and relatively homogenous subgroups or segments based on healthcare requirements can aid healthcare resource planning and the development of targeted intervention programs for a specific patient subgroup [1, 2]. With an understanding of the current and future healthcare requirements for each segment, more targeted and efficient care can be delivered for each specific patient segment. This is especially critical in Singapore with rapidly ageing population and increasing chronic disease burden [3]. Healthcare expenditure is predicted to exponentially increase from Singapore Dollars (SGD) $4 billion (USD $2.98 billion) in 2011 to SGD $12 billion (USD $8.94 billion) in 2020 [4]. Healthcare in Singapore is mainly under the responsibility of the Singapore Ministry of Health (MOH) which uses a mixed financing system that includes nationalized healthcare insurance schemes and deductions from the compulsory savings plan Central Provident Fund (CPF), for Singapore citizens and permanent residents [5]. In order to effectively deliver effective and targeted care for an ageing population and cope with increasing healthcare costs, it is crucial to have a deep understanding of population’s health characteristics and healthcare needs. Population segmentation is a critical first step in the development of effective healthcare policy because it provides policy makers with more detailed information about specific health characteristics and healthcare needs of each population segment which allows for tailored health intervention programs for different segments. This eventually leads to better policy decisions on healthcare resources allocation and planning.

There are two major approaches to population segmentation – 1) data-driven approach where segmentation is done using statistical analysis (e.g. clustering analysis, latent class analysis, classification tree) on empirical health data and 2) expert-defined approach where segments are decided via experts’ review and consensus on current evidence in literature. These two approaches are not mutually exclusive and a hybrid approach may have both data and experts input. Some examples of a data-driven approach include Lafortune’s latent class analysis of a trial’s data [6], Liu et al’s study of the Taiwan National health Insurance survey participants [7] and Van der Laan at al’s demand-driven segmentation model [8]. In these studies, health related data, including medical, behavioral, functional and socio-demographic data were used to derive various segments and profile each segment’s characteristics.

Alternatively, segments can also be defined a-priori through experts’ review and consensus on current evidence in literature. Examples of published expert-defined approaches include Lynn et al’s Bridges to Health person-centered segmentation framework, [9] Kaiser Permanente’s Senior Segmentation Algorithm for elderly persons aged 65 years or older [10] and National Academy of Medicine Patient Taxonomy [11]. In our previous work, we assessed the feasibility of segmenting a general patient population into six segments defined by Singapore Health Systems Regional Health System (SingHealth RHS) experts [12]. In our previous work, we found this framework to be feasible as a proof of concept to identify patient segments with distinct healthcare utilization and mortality patterns [12]. However, in the previous study, we were not able to assess the predicative ability of patient segment membership on long-term healthcare utilization and mortality. It is important that validation and adjustment need to be pursued before clinical and policy application in a healthcare system [13]. In our policy context, the segmentation approach needs to be validated against long-term healthcare utilization and mortality. This is also a critical gap in literature where it is not clear whether population segments by expert-defined segmentation approaches have different long-term healthcare utilization and mortality.

In this study, we aimed to address this critical gap by assessing the predictive ability of our expert defined segmentation approach on 3-year healthcare utilization (defined as hospital admissions, emergency department attendances, and specialist outpatient clinic attendances) and mortality rate.

## Methods

### Study design

We conducted a retrospective study to segment all adult patients (≥ 21 years of age in Year 2012) who utilized healthcare services at SingHealth RHS in 2012. Patients were excluded if they were below 21 years of age. This study was approved by SingHealth Centralized Institutional Review Board (CIRB 2016/2294). De-identified data from 2012 to 2015 were extracted from the electronic health records (EHRs) using the Oracle Business Intelligence and Enterprise Edition (OBIEE) Software [14]. The extracted variables included socio-demographic data, chronic diseases, healthcare utilization (hospital admissions, emergency department attendances and specialist outpatient clinic attendances) and mortality.

### Segmentation classification

A previously described segmentation framework was used [12]. The experts who developed the current framework are senior health administrators with extensive experience in both health policy and clinical care. This is to ensure policy and implementation relevance in our healthcare system setting. Patients were segmented into six non-overlapping subgroups: Mostly Healthy, Stable Chronic, Serious Acute, Complex Chronic without Frequent Hospital Admissions, Complex Chronic with Frequent Hospital Admissions, and End of Life. The definitions and examples of the segments are elaborated in Additional file 1 and Additional file 2. We defined frequent hospital admissions as 3 or more hospital admissions in past 12 months, which is a proxy for high cost users [15–18].

### Statistical analysis

We firstly compared the socio-demographics and hospital utilization in baseline year 2012 between each segment using Chi-square for categorical variables and one-way ANOVA test for continuous variables. Using the start date of 1st January 2013 as time of entry into the study for all patients, we calculated the time to survival as the number of days from entry to death (for patients who are deceased on or before 31st December 2015) or 1094 days for censored patients (number of days from entry to 31st December 2015). Kaplan-Meier survival curves were plotted and differences in the survival plots were analyzed using log-rank test. To determine if there are differences in the hospital utilization from year 2013 to 2014, we first conducted bivariate analyses between the population segment and the hospital utilization using ANOVA or Chi-square test. As the count data for the utilization rate is over-dispersed where most of the patients actually have 0 utilization, a negative binomial regression model was used to model the hospital utilization with the Mostly Healthy segment as the reference group for the segments, and adjusted for age, gender, and ethnicity and past hospital utilization. We used the survival time as the exposure variable for the negative binomial regression model. We also conducted two-degree freedom Chi-square test between each pair of segments to test for significant difference of hospital utilization. All analyses were performed on STATA/IC 13.1.

## Results

### Patient baseline characteristics and acute hospital utilization

A total of 819,993 patients were included and segmented into the six segments with the proportions shown in Table 1. The overall mean age of the study population was 49.8 years with standard deviation (SD) of 17.2. There are more female than male patients (58% vs. 42%). The differences in age and gender between the segments are statistically significant with *p* < 0.001.

**Table 1:** Demographics and Healthcare Utilization of Patients by Segments in Baseline Year 2012

Demographics	Mostly Healthy	Serious Acute	Stable Chronic	Complex Chronic without Frequent Hospital Admissions	Complex Chronic with Frequent Hospital Admissions	End of Life	All	*p*-value^a^
Total (%)	481,772 (58.8%)	200,925 (24.5%)	43,757 (5.3%)	87,632 (10.7%)	3935 (0.5%)	1972 (0.2%)	819,993 (100.0%)	
Age (in years)								
Mean (SD)	43.9 (15.6)	60.6 (13.9)	42.3 (15.8)	60.8 (15.0)	63.5 (14.5)	58.0 (13.1)	49.8 (17.2)	< 0.001
21–30 (%)	119,741 (24.9%)	6439 (3.2%)	11,932 (27.3%)	3660 (4.2%)	102 (2.6%)	56 (2.8%)	141,930 (17.3%)	< 0.001
31–40 (%)	104,275 (21.6%)	9602 (4.8%)	12,832 (29.3%)	5229 (6.0%)	186 (4.7%)	124 (6.3%)	132,248 (16.1%)	
41–50 (%)	95,434 (19.8%)	25,956 (12.9%)	6686 (15.3%)	10,914 (12.5%)	402 (10.2%)	347 (17.6%)	139,749 (17.0%)	
51–60 (%)	84,221 (17.5%)	54,330 (27.0%)	5524 (12.6%)	21,110 (24.1%)	867 (22.0%)	598 (30.3%)	166,640 (20.3%)	
61–70 (%)	49,255 (10.2%)	54,825 (27.3%)	3754 (8.6%)	21,970 (25.1%)	1003 (25.5%)	500 (25.4%)	131,307 (16.0%)	
71–80 (%)	22,399 (4.6%)	36,246 (18.0%)	2161 (4.9%)	17,423 (19.9%)	937 (23.8%)	264 (13.4%)	79,430 (9.7%)	
81–90 (%)	5878 (1.2%)	12,184 (6.1%)	750 (1.7%)	6509 (7.4%)	380 (9.7%)	74 (3.8%)	25,775 (3.1%)	
> 90 (%)	569 (0.1%)	1343 (0.7%)	108 (0.2%)	827 (0.9%)	58 (1.5%)	9 (0.5%)	2914 (0.4%)	
Gender								
Male (%)	197,774 (41.1%)	90,856 (45.2%)	12,466 (28.5%)	40,581 (46.3%)	2017 (51.3%)	809 (41.0%)	344,503 (42.0%)	< 0.001
Ethnicity								
Chinese (%)	328,982 (68.3%)	154,035 (76.7%)	25,425 (58.1%)	66,829 (76.3%)	2821 (71.7%)	1503 (76.2%)	579,595 (70.7%)	< 0.001
Malay (%)	47,270 (9.8%)	24,439 (12.2%)	6992 (16.0%)	8864 (10.1%)	526 (13.4%)	157 (8.0%)	88,248 (10.8%)	
Indian (%)	43,413 (9.0%)	14,218 (7.1%)	5100 (11.7%)	7084 (8.1%)	407 (10.3%)	82 (4.2%)	70,304 (8.6%)	
Others (%)	62,107 (12.9%)	8233 (4.1%)	6240 (14.3%)	4855 (5.5%)	181 (4.6%)	230 (11.7%)	81,846 (10.0%)	
ED visits in 2012								
Median (IQR)	0 (0–0)	0 (0–0)	0 (0–0)	0 (0–1)	3 (1–4)	0 (0–1)	0 (0–0)	< 0.001
Yes (%)	20,206 (4.2%)	13,386 (6.7%)	3004 (6.9%)	16,786 (19.2%)	1998 (50.8%)	345 (17.5%)	55,725 (6.8%)	< 0.001
SOC visits in 2012								
Median (IQR)	1 (0–3)	0 (0–2)	6 (3–12)	5 (2–11)	20 (12–33)	23 (9–45)	1 (0–4)	< 0.001
Yes (%)	199,246 (41.4%)	81,491 (40.6%)	20,443 (46.7%)	64,523 (73.6%)	3149 (80.0%)	1438 (72.9%)	370,290 (45.2%)	< 0.001
Hospital Admissions in 2012								
Median (IQR)	0 (0–0)	0 (0–0)	1 (1–1)	0 (0–1)	4 (3–5)	1 (0–2)	0 (0–0)	< 0.001
Yes (%)	17,258 (3.6%)	10,187 (5.1%)	4191 (9.6%)	18,760 (21.4%)	2154 (54.7%)	584 (29.6%)	53,134 (6.5%)	< 0.001

*Abbreviations*: *ED* Emergency Department, *SOC* Specialist Outpatient Clinic. Numbers were presented as median ± interquartile range or number (%) as appropriate

^a^ Chi-square for categorical variables and one-way ANOVA test for continuous variables

There is a trend of increasing hospital utilization in 2012 as we moved down the segments from Mostly Healthy to Complex Chronic with Frequent Hospital Admissions (Table 1). The differences between the six segments are all statistically significant with *p* < 0.001 for ED visits, SOC visits and hospital admissions. Not unexpectedly, patients in the Complex Chronic with Frequent Admissions segment had more frequent admissions, as this was a criterion for inclusion in this segment. However, this pattern of increased utilization in this group was also seen for SOC and ED attendances, suggesting that this segment does have increased healthcare utilization in multiple areas.

### Bivariate analyses of segments and hospital utilization from year 2013 to 2015

The trend that we observed for hospital utilization from year 2013 to 2015 is similar to the trend for hospital utilization in 2012 where there is an increasing number of ED visits, SOC visits and hospital admissions from the Mostly Healthy segment to the Complex Chronic with Frequent Hospital Admissions (Table 2). Patients in the End of Life segment had the most SOC visits (mean 43.2, SD 50.8) among all six segments but they had significantly less ED visits (mean 0.88, SD 1.67) and hospital admissions (mean 1.33, SD 2.10) than patients in the Complex Chronic with Frequent Hospital Admissions segment (mean 4.00, SD 7.29 for ED visit; mean 4.49, SD 6.32 for hospital admissions). The hospital utilization is significantly different for the six segments with *p* < 0.001.

**Table 2:** Bivariate Analyses of Segments versus Hospital Utilization Rates from Year 2013 to 2015

Outcome	Mostly Healthy	Serious Acute	Stable Chronic	Complex Chronic without Frequent Hospital Admissions	Complex Chronic with Frequent Hospital Admissions	End of Life	All	*p*-value
Total no. of patient days	525,336,500	218,597,459	47,521,470	91,359,758	3,320,804	1,548,823	887,684,814	
Total ED visit (2013 to 2015)								
Grand Total	70,679	58,964	15,050	75,126	15,734	1726	237,279	
No. per 1000 patient days	0.13	0.27	0.32	0.82	4.74	1.11	0.27	
Mean (SD)	0.15 (0.62)	0.29 (0.99)	0.34 (1.34)	0.86 (2.25)	4.00 (7.29)	0.88 (1.67)	0.88 (1.22)	< 0.001
Visited ED (%)	44,065 (9.2%)	31,249 (15.6%)	7121 (16.3%)	28,105 (32.1%)	2808 (71.4%)	780 (39.6%)	114,128 (13.9%)	< 0.001
Total SOC visit (2013 to 2015)								
Grand Total	2,670,226	1,417,950	411,584	1726,201	167,529	85,152	6,478,642	
No. per 1000 patient days	5.08	6.49	8.66	18.89	50.45	54.98	7.30	
Mean (SD)	5.54 (10.88)	7.06 (14.15)	9.41 (14.94)	19.70 (26.85)	42.57 (42.69)	43.18 (50.75)	7.90 (15.80)	< 0.001
Visited SOC (%)	273,616 (56.8%)	115,599 (57.5%)	32,183 (73.6%)	73,789 (84.2%)	3823 (97.2%)	1768 (89.7%)	500,778 (61.1%)	< 0.001
Total Hospital Admissions (2013 to 2015)								
Grand Total	64,766	47,723	19,696	71,711	17,674	2620	224,190	
No. per 1000 patient days	0.12	0.22	0.41	0.78	5.32	1.69	0.25	
Mean (SD)	0.13 (0.54)	0.24 (0.84)	0.45 (1.09)	0.82 (1.90)	4.49 (6.32)	1.33 (2.10)	0.27 (1.06)	< 0.001
Admitted to Hospital (%)	43,025 (8.9%)	26,568 (13.2%)	11,350 (25.9%)	27,792 (31.7%)	3062 (77.8%)	985 (50.0%)	112,782 (13.8%)	< 0.001

*Abbreviations*: *ED* Emergency Department, *SOC* Specialist Outpatient Clinic. Numbers were presented as mean ± standard deviation or number (%) as appropriate

^a^ Chi-square for categorical variables and one-way ANOVA test for continuous variables

### Multivariable negative binomial regression on hospital utilization from year 2013 to 2015

As compared to the Mostly Healthy segment, patients in all other segments have significantly higher ED visits (*p* < 0.001) after adjusting for age, gender, ethnicity and hospital utilization in year 2012 (Table 3). Patients in the Complex Chronic with Frequent Admissions segment have 14.5 times (95% Confidence Interval: 13.49–15.64) ED visits compared to patients in the Mostly Healthy segment. Patients in the End of Life segment also have a highly increased risk of having ED visits compared to patients in the Mostly Healthy segment with an incident rate ratio (IRR) of 9.56 (95% CI: 8.51–10.75).

**Table 3:** Multivariable Negative Binomial Regression on Hospital Utilization from Year 2013 to 2015

	IRR	95% Confidence Interval	*p*-value
No. of ED visits from 2013 to 2015			
Mostly Healthy	*Reference*		
Serious Acute	1.45	(1.42, 1.47)	< 0.001
Stable Chronic	2.15	(2.09, 2.21)	< 0.001
Complex Chronic without Frequent Hospital Admissions	3.36	(3.29, 3.44)	< 0.001
Complex Chronic with Frequent Hospital Admissions	14.52	(13.49, 15.64)	< 0.001
End of Life	9.56	(8.51, 10.75)	< 0.001
No. of SOC visits from 2013 to 2015			
Mostly Healthy	*Reference*		
Serious Acute	1.03	(1.02, 1.04)	< 0.001
Stable Chronic	1.91	(1.88, 1.94)	< 0.001
Complex Chronic without Frequent Hospital Admissions	2.67	(2.64, 2.71)	< 0.001
Complex Chronic with Frequent Hospital Admissions	7.71	(7.31, 8.13)	< 0.001
End of Life	11.50	(10.68, 12.39)	< 0.001
No. of Hospital Admissions from 2013 to 2015			
Mostly Healthy	*Reference*		
Serious Acute	1.33	(1.31, 1.36)	< 0.001
Stable Chronic	3.16	(3.08, 3.25)	< 0.001
Complex Chronic without Frequent Hospital Admissions	3.86	(3.78, 3.94)	< 0.001
Complex Chronic with Frequent Hospital Admissions	22.66	(21.07, 24.37)	< 0.001
End of Life	16.18	(14.49, 18.07)	< 0.001

*Abbreviations*: *ED* Emergency Department, *SOC* Specialist Outpatient Clinic, *IRR* Incidence Rate Ratio. Model is adjusted for age, gender, ethnicity, past utilization and use of survival time as exposure variables

For SOC, compared with Mostly Healthy segment, all the other segments have significantly higher utilization than the (all *p* < 0.001). After adjusting for the baseline variables and hospital utilization in 2012, patients in the End of Life segment have 11.50 times (95% CI: 10.68–12.39) SOC utilization compared to patients in the Mostly Healthy segment. Patients in the Complex Chronic with Frequent Admissions segment also have a significantly higher utilization than patients in the Mostly Healthy segment (IRR 7.71, 95% CI: 7.31–8.13).

Lastly, compared to the Mostly Healthy segment, all other segments have significantly high inpatient admissions with IRRs > 1 (*p* < 0.001). Patients in the Complex Chronic with Frequent Admissions segment had the highest IRR of 22.66 (95% CI: 21.07–24.37) for hospital admissions from 2013 to 2015. Patients in the End of Life segment have the second highest IRR of 16.18 (95% CI: 14.49–18.07).

For each model, the Chi-square tests showed that there are significant differences between all pair-wise segments with *p* < 0.001.

### Analysis of survival time

Day 0 was taken at 1st January 2013. At the end of 2013, the survival rates for patients in the End of Life and Complex Chronic with Frequent Hospital Admissions segments were 74.6 and 81.7% respectively, while the survival rates for Complex Chronic without Frequent Hospital Admissions, Stable Chronic, Serious Acute and Mostly Healthy segments were all > 95%.

At the end of the second year (2014), the survival rates for patients in the End of Life and Complex Chronic with Frequent Hospital Admissions segments were 64.6 and 71.0% respectively, while the survival rates for Complex Chronic without Frequent Hospital Admissions, Stable Chronic, Serious Acute and Mostly Healthy segments were all > 93%.

Overall, patients in the End of Life segment had the worst survival rate (58.2%), followed by patients in the Complex Chronic with Frequent Hospital Admissions (62.6%) at the end of 3 years (end of 2015). Throughout the 3 years 2013–2015, the survival rates for patients in the Mostly Healthy, Serious Acute and Stable Chronic segments were indistinguishable from each other and higher than the other three segments (Additional file 3). The log-rank test for equality of the six survival distributions showed statistically significant difference between the six segments (*p* < 0.001).

## Discussion

Our study supports that our previously developed six-segment framework is predictive of long-term healthcare utilization and mortality. Healthcare utilization and mortality increased with the complexity of the segments, suggesting that our segmentation approach was able to discriminate between patients of varying healthcare needs and risk of mortality. Patients in the Complex Chronic with Frequent Hospital Admissions segment represented 0.5% of the study population, but accounted for the highest risk of hospital admissions and ED visits per patient, and second highest risk of SOC visits in the following 3 years (2013–2015) after the initial healthcare encounter in 2012. Moreover, about one in three patients in this segment died within the next 3 years. This suggests that patients in segment had high healthcare burden that requires further investigation into disease management, psychosocial environment and quality of community care within the segment. Equally worth noting is the End of Life segment that accounted for highest SOC visits. This is likely due to the nature of patients within the End of Life segment – many of them have metastatic cancer with frequent outpatient appointments.

For the Mostly Healthy, Serious Acute, and Stable Chronic segments, survival rates were similar from 2013 to 2015 although there was an increasing gradient of healthcare utilization over the same period of time. This is important information in population health management which does not only consider survival but also healthcare resource consumptions and service planning. In a healthcare system where increasing healthcare spending is of particular concern, healthcare resource consumption trends are relevant and of particular interest to our policy makers.

There are several strengths of our approach. Firstly, this simple categorization can be easily replicated in most healthcare systems as the variables and healthcare utilization measures used in our study are commonly available in other healthcare systems. Some of the recently implemented segmentation framework such as those used in British Columbia, Canada [19] and Northern London, UK [20] used similar domains of information as our framework. While our study successfully identified six distinct segments with different long-term healthcare utilization and mortality, we are cognizant that even within each segment, patients may have differing healthcare needs. The utility of the current segmentation approach is less about specific disease treatment for a specific patient over a single healthcare encounter, which requires individualization of management plan by each patient-healthcare provider pair, but more relevant at policy level in planning what types of health services are needed for each segment at population level. Our segmentation framework is practical, with each segment corresponding to a predominant site of care and bundle of interventions. For example, subjects in the Mostly Healthy and the Serious Acute segments require mainly community-based health promotion activities and lifestyle interventions. This will guide population health policy and lead to more resources in preventive services development and health promotion efforts. Patients in the Stable Chronic segment require mainly primary care to avoid progression to complications while patients in the Complex Chronic with Frequent Hospital Admissions segment and Complex Chronic without Frequent Hospital Admissions segment may benefit from more aggressive and multi-disciplinary services for case management. For the End of Life segment, hospice care is typically needed to manage symptoms and to avoid events such as unnecessary hospitalizations that may be expensive and potentially risky. By knowing there is an End of Life segment and what is the proportion of entire patient population that belong to this segment, healthcare policy makers can allocate appropriate health resources in developing advanced care plans and shared care with appropriate specialists and/or team-based care, community case coordinators to optimize quality of life.

Our study has several limitations. First, variables in our dataset were restricted to those routinely collected in our EHRs. We were hence unable to refine the segmentation using information on functional status and socioeconomic variables which play important roles in influencing health related behavior and health services utilizations [21]. Secondly, our population database is unable to account for cross-utilization of healthcare services outside of the SingHealth or out of hospital deaths.

Data-driven segmentation approaches also provide an attractive alternative to generating evidence-based insights of a population’s health status. These approaches include unsupervised techniques such as clustering analysis and latent class analysis, and supervised techniques such as classification and regression. A key strength of data-driven approaches is the potential to group similar patients according to their similarity in several dimensions or characteristics [22]. Non-apparent latent classes or clusters can then be identified based on similar characteristics. Data-driven frameworks, although easy to standardize and explicit in methodology, may not always be relevant and practical at policy and implementation level in a particular healthcare system. Experts driven methods are likely to have implementation feasibility and policy implications but may not have the rich insights from large volume of health data. It is each healthcare system’s decision to adopt either experts-driven, data-driven, or a hybrid approach taking into considerations scientific evidence and specific policy contexts and priorities.

## Conclusion

In this study, we demonstrated the predictive ability of an expert-driven segmentation framework on longitudinal healthcare utilization and mortality.

## Additional files

Additional file 1: Definitions of the Population Segments (DOCX 16 kb)

Additional file 2: Classification of Chronic Diseases (DOCX 15 kb)

Additional file 3: Kaplan-Meier Survival Estimate by Patient Segment (DOCX 215 kb)

## Data Availability

The dataset used for this study can be found at Harvard Dataverse: https://dataverse.harvard.edu/dataset.xhtml?persistentId=doi:10.7910/DVN/XTXCYD.

## Abbreviations

CPF: Central Provident Fund
ED: Emergency Department
IRR: Incidence Rate Ratio
MOH: Ministry of Health
SD: Standard Deviation
SES: Socioeconomic Status
SingHealth: Singapore Health Services
SOC: Specialist Outpatient Clinic
