# Supplementary material for: Predictive ability of an expert-defined population segmentation framework for healthcare utilization and mortality - a retrospective cohort study
Source: BMC Health Serv Res. 2019 Jun 20;19:401. doi: 10.1186/s12913-019-4251-6 (PMC6585096; doi:10.1186/s12913-019-4251-6)
Supplement: Supplementary file 1 — Definitions of the Population Segments (DOCX 16 kb) [file 12913_2019_4251_MOESM1_ESM.docx]

Table S1: Definitions of the Population Segments

| **Category** | **Definition** | **Descriptive Examples** |
| --- | --- | --- |
| Mostly Healthy | No chronic diseases | A patient who saw a primary care physician for upper respiratory tract infection or attended the emergency department for gastritis. |
| Stable Chronic | At least 1 chronic disease (that does not interfere with/restrict normal function or sufficient to trigger care seeking)** and 0 related admissions in one year | A patient who saw a primary care physician for hypertension. |
| Serious Acute | No chronic diseases but had admissions for acute conditions e.g. acute appendicitis. Primary discharge diagnosis not related to defined chronic conditions in Supplementary table 2. | A patient who was admitted to hospital for acute appendicitis. |
| Complex Chronic without Frequent Hospital Admissions | At least 1 chronic disease* (that interfere with/restrict normal function or sufficient to trigger care seeking)** and <3 related admissions in one year | A patient with hypertension, hyperlipidemia, atrial fibrillation, rheumatoid arthritis on follow up with hospital. |
| Complex Chronic with Frequent Hospital Admissions | At least 1 chronic disease* (that interfere with/restrict normal function)** and recurrent admissions i.e. ≥3 in one year | A heart failure patient with frequent hospital admissions for fluid overload. |
| End of Life | Metastatic disease | A patient with metastatic cancer. |
