# Supplementary material for: Predictive ability of an expert-defined population segmentation framework for healthcare utilization and mortality - a retrospective cohort study
Source: BMC Health Serv Res. 2019 Jun 20;19:401. doi: 10.1186/s12913-019-4251-6 (PMC6585096; doi:10.1186/s12913-019-4251-6)
Supplement: Supplementary file 2 — Classification of Chronic Diseases (DOCX 15 kb) [file 12913_2019_4251_MOESM2_ESM.docx]

Table S2: Classification of Chronic Diseases

| **Stable Chronic Diseases** |
| --- |
| 1. Diabetes without chronic complication |
| 2. Hypertension |
| 3. Chronic kidney disease without End Stage Renal Failure |
| 4. Asthma |
| 5. Hyperlipidemia |
| 6. Osteoarthritis |
| 7. Osteoporosis |
| 8. Benign Prostatic Hypertrophy |
| 9. Chronic Obstructive Pulmonary Disease without cor pulmonale |
| 10. Hyperthyroidism |
| 11. Hypothyroidism |
|  |
| **Complex Chronic Diseases*** |
| 1. Diabetes with chronic complications |
| 2. Cerebrovascular disease |
| 3. Chronic Kidney Disease Stage 5 or End Stage Renal Failure |
| 4. Chronic Obstructive Pulmonary Disease with cor pulmonale |
| 5. Major depression |
| 6. Schizophrenia |
| 7. Dementia |
| 8. Bipolar disorder |
| 9. Collagen Vascular diseases |
| 10. Anxiety |
| 11. Parkinson’s disease |
| 12. Epilepsy |
| 13. Coronary heart disease, myocardial infarction |
| 14. Atrial fibrillation |
| 15. Hip fracture |
| 16. Spine fracture |
| 17. Moderate or severe liver disease, Liver cirrhosis |
| 18. Any malignancy, non-metastatic |
| 19. Thromboembolism: prosthetic valve, thrombosis, embolism |
| 20. Pressure Ulcer |
| 21. Heart failure and Fluid overload |
| 22. Peripheral vascular disease |
|  |
| **End of life** |
| 1. Metastatic disease |

* A complex chronic disease is defined as one that that interfered with / restricted normal function or was sufficient to trigger care seeking
