# Supplementary material for: Predictive ability of an expert-defined population segmentation framework for healthcare utilization and mortality - a retrospective cohort study
Source: BMC Health Serv Res. 2019 Jun 20;19:401. doi: 10.1186/s12913-019-4251-6 (PMC6585096; doi:10.1186/s12913-019-4251-6)
Supplement: Supplementary file 3 — Kaplan-Meier Survival Estimate by Patient Segment (DOCX 215 kb) [file 12913_2019_4251_MOESM3_ESM.docx]

Figure S1. Kaplan-Meier Survival Estimate by Patient Segment

|  |  |  |  |  |  |  |  |  |  |  |  |
| --- | --- | --- | --- | --- | --- | --- | --- | --- | --- | --- | --- |
| Interval (days) | | 0 | 120 | 240 | 360 | 480 | 600 | 720 | 840 | 960 | 1080 |
| Number at Risk | | | | | | | | | | | |
| Mostly Healthy | | 481772 | 481457 | 481130 | 480812 | 480470 | 480080 | 479737 | 479310 | 478865 | 478477 |
| Serious Acute | | 200925 | 200743 | 200526 | 200291 | 200023 | 199748 | 199463 | 199147 | 198839 | 198542 |
| Stable Chronic | | 43757 | 43683 | 43618 | 43548 | 43472 | 43406 | 43341 | 43275 | 43197 | 43130 |
| Complex Chronic without Frequent Hospital Admissions | | 87632 | 86596 | 85684 | 84765 | 83923 | 83007 | 82215 | 81450 | 80651 | 79933 |
| Complex Chronic with Frequent Hospital Admissions | | 3935 | 3595 | 3392 | 3215 | 3058 | 2910 | 2799 | 2676 | 2557 | 2472 |
| End of Life | | 1972 | 1733 | 1591 | 1473 | 1400 | 1333 | 1275 | 1234 | 1199 | 1151 |
